# Associations between legal performance-enhancing substance use and future cardiovascular disease risk factors in young adults: A prospective cohort study

**DOI:** 10.1371/journal.pone.0244018

**Published:** 2020-12-15

**Authors:** Jason M. Nagata, Kyle T. Ganson, Mitchell L. Cunningham, Deborah Mitchison, Jason M. Lavender, Aaron J. Blashill, Holly C. Gooding, Stuart B. Murray

**Affiliations:** 1 Department of Pediatrics, University of California, San Francisco, San Francisco, California, United States of America; 2 Factor-Inwentash Faculty of Social Work, University of Toronto, Toronto, Ontario, Canada; 3 School of Psychology, University of Sydney, Sydney, New South Wales, Australia; 4 Translational Health Research Institute, School of Medicine, Western Sydney University, Sydney, New South Wales, Australia; 5 Department of Psychology, Macquarie University, Sydney, New South Wales, Australia; 6 Military Cardiovascular Outcomes Research Program (MiCOR), Department of Medicine, Uniformed Services University of the Health Sciences, Bethesda, Maryland, United States of America; 7 The Metis Foundation, San Antonio, Texas, United States of America; 8 Department of Psychology, San Diego State University, San Diego, California, United States of America; 9 San Diego Joint Doctoral Program in Clinical Psychology, San Diego State University, San Diego, California, United States of America; 10 San Diego Joint Doctoral Program in Clinical Psychology, University of California, San Diego, California, United States of America; 11 Department of Pediatrics, Emory University School of Medicine, Atlanta, Georgia, United States of America; 12 Department of Psychiatry & Behavioral Sciences, University of Southern California, Los Angeles, California, United States of America; McMaster University, CANADA

## Abstract

**Background:**

Legal performance-enhancing substances (PES), such as creatine, are commonly used by adolescents and young adults. As PES are mostly unregulated by the US Food and Drug Administration, there has been limited empirical attention devoted to examining their long-term safety and health outcomes. Preliminary studies have demonstrated associations between PES use and severe medical events, including hospitalizations and death. PES could be linked to cardiovascular disease (CVD), the most common cause of mortality in the US, by altering the myocardium, vasculature, or metabolism. The objective of this study was to examine prospective associations between the use of legal PES in young adulthood and CVD risk factors at seven-year follow-up.

**Materials and methods:**

Nationally representative longitudinal cohort data from the National Longitudinal Study of Adolescent to Adult Health, Waves III (2001–2002) and IV (2008), were analyzed. Regression models determined the prospective association between the use of legal PES (e.g. creatine monohydrate) and CVD risk factors (e.g. body mass index, diabetes, hypertension, hyperlipidemia), adjusting for relevant covariates.

**Results:**

Among the diverse sample of 11,996 male and female participants, no significant differences by PES use in body mass index, diabetes, hypertension, or hyperlipidemia were noted at Wave III. In unadjusted comparisons, legal PES users (versus non-users) were more likely to be White, be male, be college educated, drink alcohol, and engage in weightlifting, exercise, individual sports, team sports, and other strength training. There were no significant prospective associations between legal PES use at Wave III and body mass index, hemoglobin A1c, systolic and diastolic blood pressure, and cholesterol (total, HDL, LDL, triglycerides) deciles at seven-year follow-up (Wave IV), adjusting for demographics, health behaviors, and Wave III CVD risk factors. Similarly, there were no significant prospective associations between legal PES use and diabetes, hypertension, or hyperlipidemia based on objective measures or self-reported medications and diagnoses, adjusting for demographics, health behaviors, and Wave III CVD risk.

**Conclusions:**

We do not find evidence for a prospective association between legal PES use and CVD risk factors in young adults over seven years of follow-up, including BMI, diabetes, hypertension, or hyperlipidemia. It should be noted that legal PES use was operationalized dichotomously and as one broad category, which did not account for frequency, amount, or duration of use. Given the lack of regulation and clinical trials data, observational studies can provide much needed data to inform the safety and long-term health associations of legal PES use and, in turn, inform clinical guidance and policy.

## Introduction

Performance-enhancing substances (PES) are commonly used among adolescents and young adults and are associated with increased health risks [1, 2]. In the United States (US), PES can be delineated by legal status, where legal PES include substances such as creatine monohydrate and amino acids and illegal PES include anabolic-androgenic steroids (AAS) not prescribed for medical purposes. Research on AAS misuse has documented adverse social, psychological [3–6], and physiological [7–9] health outcomes, although AAS are less commonly used (3–6% among boys and men) compared to legal PES [1, 2]. In contrast, between 16–35% of adolescent and young adult males in the US have used legal PES in the past year [1, 2], with easy accessibility and lack of regulatory oversight of legal PES likely contributing to high use rates. Legal PES use has also been found to be prospectively associated with AAS misuse [10]. Notably, a recent study in young people documented that legal PES use, including muscle-enhancing or energy supplements, was associated with an increased risk of severe medical events (e.g., emergency room visits, disability, or premature death) when compared to vitamin use [11]. Unfortunately, there has been a dearth of research investigating health-related outcomes associated with legal PES use over the long term [3].

Cardiovascular health outcomes represent an area in particular need of investigation, given that many cardiovascular disease (CVD) risk factors (e.g., diabetes, hypertension, dyslipidemia) commonly develop in young adulthood when legal PES use is prevalent, and CVD remains the leading cause of death in the US [12, 13]. Legal PES may have effects on the cardiovascular system by directly altering the myocardium, vasculature, or metabolism [14]. Furthermore, there is the potential for indirect effects by enabling users to push beyond normal physiologic limits with potential consequences of exercise-induced arrhythmias [14]. PES use is associated with eating disorders and muscle dysmorphia [15–17], which have cardiovascular consequences [18, 19]. These potential cardiovascular effects warrant further examination, especially given mixed findings in various short-term health-related outcomes. For example, short-term creatine supplementation is generally safe in adults [20], does not appear to adversely affect blood lipid profiles (e.g., cholesterol, triglycerides) [21], and may even have favorable glycemic effects (when combined with exercise) for individuals with type-2 diabetes [22]. However, creatine use also has been linked to weight gain [23], which could represent a detriment to long-term cardiovascular health, as higher weight and BMI are primary risk factors for CVD [24]. Additionally, recent reviews have found that stimulants are associated with significant elevations in systolic and diastolic blood pressure [25, 26]. Caffeine, a stimulant that can be used for performance enhancement and is commonly included in mixtures of legal PES, activates the sympathetic nervous system and catecholamines, leading to elevations in blood pressure [27, 28]. Finally, other legal forms of PES, such as androgen prohormones, could have particularly deleterious health-related impacts, especially if associated with adverse cardiovascular effects similar to those resulting from misuse of AAS (which they are designed to chemically emulate) [20].

Importantly, legal PES are often mislabeled and inaccurate in terms of their contents [11, 29, 30], which may explain serious medical outcomes that can be associated with their use. Specifically, there are concerns that a significant number of dietary supplementary products can be adulterated with potent stimulants (e.g., derivatives of methamphetamine) and other illicit substances (e.g., AAS) [31–34] with serious cardiovascular effects [14]. For instance, 15–60% of nutritional supplements were found to contain AAS when tested [35, 36]. A recent review found a link between AAS and CVD risk factors, including hypertension or dyslipidemia, in seven of the nine studies reviewed [37], consistent with prior reviews [38–41]. Potential mechanisms underlying this link include increased systemic inflammation, oxidative stress, and vascular dysfunction [41]. Consequently, it is plausible that use of a legal PES may promote CVD risk by virtue of the unlabeled ingredients or contaminants they may contain. Furthermore, amount, frequency, and duration of use are likely to be factors related to adverse outcomes. For example, high doses and prolonged use of AAS have been found to be associated with greater degrees of dyslipidemia [41].

In sum, although preliminary research points to a prospective link between legal PES use and severe medical complications, there is a need to investigate long-term, prospective associations with specific health outcomes. In the present study, we focus on CVD risk factors–including diabetes, hypertension, abnormal lipid profiles, and obesity–in young adulthood given that these outcomes may take many years to fully manifest [38, 42] and evidence suggesting that use of illegal PES (e.g., AAS) is associated with CVD risk factors [37–41]. Additionally, previous studies have been limited by small and/or highly specific samples (e.g., athletes) or comparison groups (e.g., vitamin users), warranting further evidence that large sample, population-based cohort studies are needed. Thus, the objective of this investigation was to examine the prospective association between legal PES use in young adulthood and several key CVD risk factors, using data collected in a nationally representative longitudinal cohort study.

### Materials and methods

#### Study population

This study utilizes data from the National Longitudinal Study of Adolescent to Adult Health (Add Health). Add Health is a nationally representative longitudinal cohort study which sampled adolescents throughout the United States and followed them into adulthood [43]. The baseline sample data was collected from 1994 to 1995 when participants were 11–18 years old by using systematic sampling methods and implicit stratification so that the selected high schools (n = 80) and paired middle schools were representative of US schools with respect to geographical region of the country, size, urbanicity, type (i.e. public, private, charter), and race/ethnicity. For this specific study, we used the nationally representative, restricted-use samples from Wave III (18–26 years, 2001–2002) and Wave IV (24–32 years, 2008). At Waves III and IV, data were collected via interview, lasting approximately 90 minutes, at the participant’s home or other suitable location. Immediately following the interview, interviewers took physical measurements and collected biological specimens. The University of North Carolina Institutional Review Board approved all Add Health study procedures and written informed consent was obtained. Further details about the study design can be found elsewhere [43].

#### Measures

*Predictor*. The primary predictor variable of this study (asked at Wave III) was based on the question: “In the past year, have you used a legal performance enhancing substance for athletes (such as creatine monohydrate or andro)?” Response choices included “yes” or “no.”

*Outcomes*. The primary outcome measures of this study are listed below and included measurements of several CVD risk factors, such as body mass index (BMI), hemoglobin A1c, blood pressure, and cholesterol at Wave IV.

*Body mass index (BMI)*. was calculated using the standard formula weight (kilograms) divided by height (meters) squared (BMI = weight/height^2^). Weight (Health-o-meter 844KL High Capacity Digital Bathroom Scale; Jarden Corporation; Rye, NY) and height (Carpenter’s square, steel tape measure) were measured by the interviewer. Change in BMI was calculated as Wave IV BMI minus Wave III BMI (e.g., positive values indicate BMI gain).

*Hemoglobin A1c*. We examined hemoglobin A1c in Wave IV as a continuous outcome. Respondents in the study were also classified as having diabetes in Wave IV if they had levels of fasting glucose ≥ 126 mg/dl, non-fasting glucose ≥ 200 mg/dl, and hemoglobin A1c ≥ 6.5% [44], had a self-reported history of diabetes (except during pregnancy), or if they used anti-diabetic medication in the four weeks preceding the Wave IV assessment. We included self-reported diagnosis of diabetes at Wave III as a covariate.

*Blood pressure*. We examined systolic and diastolic blood pressure as continuous outcomes, using the mean of two measurements separated by a 30 second interval from a factory-calibrated, Microlife BP3MC1-PC-IB oscillometric blood pressure monitor (MicroLife USA, Inc.; Dunedin, FL). Hypertension was defined as a measured systolic blood pressure ≥130 mmHg or a measured diastolic blood pressure ≥80 mmHg [45], the use of anti-hypertensive medication in the four weeks preceding the Wave IV assessment, or an affirmative response to the query: “Have you ever been diagnosed with high blood pressure or hypertension?” In 2017, the American College of Cardiology/American Heart Association Task Force on Clinical Practice Guidelines lowered the threshold for systolic and diastolic blood pressure to define hypertension (130/80 from 140/90), expanding the number of young adults with hypertension [45]. This choice was based on meta-analyses of observational studies that demonstrated that elevated blood pressure and hypertension are associated with increased risk of CVD, end stage renal disease, subclinical atherosclerosis, and all-cause death [45]. We included self-reported diagnosis of high blood pressure or hypertension at Wave III as a covariate.

*Cholesterol*. Total cholesterol, high-density lipoprotein (HDL) cholesterol, low-density lipoprotein (LDL) cholesterol, and triglyceride deciles were defined as continuous outcomes for this study. Hyperlipidemia was defined as the total cholesterol decile corresponding to the proportion of young adults with a total cholesterol ≥240 mg/dL in the nationally representative National Health and Nutrition Examination Surveys [12, 46], the use of antihyperlipidemic medication in the four weeks preceding the Wave IV assessment, or an affirmative response to the question: “Have you ever been diagnosed with high cholesterol, triglycerides, or lipids?” We included self-reported diagnosis of high cholesterol, triglycerides, or lipids at Wave III as a covariate.

*Additional covariates*. Included self-reported demographics such as age, sex, race/ethnicity, highest education (high school or less versus college or more), and household income, as these have been shown to be associated with both legal PES use [47] and CVD risk [48]. We evaluated household income as a covariate with data from parents’ self-report of household income in the last calendar year. Gaussian normal regression imputation models were used to impute the income for the 1,638 parents who did not respond, were uncomfortable answering the income question, or stated that they did not know, similar to the method used in previous studies [2]. Behavioral covariates that are associated with legal PES use and CVD risk [12, 47, 49] were assessed at Wave III, including self-reported alcohol use (≥2 days in the past month, yes/no), smoking (past 30 days, yes/no), AAS use (between Wave I and III, yes/no), and physical activity and sports context, including weight lifting to bulk up in the past 7 days (yes/no), exercising to bulk up in the past 7 days (yes/no), the number of times participating in individual sports (running, wrestling, swimming, cross-country skiing, cycle racing, or martial arts) in the past 7 days (0–7), the number of times participating in strenuous team sports (football, soccer, basketball, lacrosse, rugby, field hockey, ice hockey) in the past 7 days (0–7), and the number of times participating in gymnastics, weight lifting, or strength training in the past 7 days (0–7).

### Statistical analysis

Data analysis was performed using Add Health’s pre-constructed sample weights to provide a sample that was nationally representative [50]. Comparisons of descriptive characteristics between legal PES users versus non-users of legal PES were calculated using Pearson’s Chi-square tests for categorical variables and independent sample *t*-tests for continuous variables. Multiple linear regression models were used to evaluate the association between legal PES use (Wave III) and continuous CVD risk factors, including BMI, BMI change, hemoglobin A1c, systolic and diastolic blood pressure, total cholesterol, LDL cholesterol, HDL cholesterol, and triglyceride deciles (Wave IV). Multiple logistic regressions were used to evaluate the association between legal PES use (Wave III) and subsequent dichotomous clinical CVD risk factors, including diabetes, hypertension, hyperlipidemia, and obesity (Wave IV). For each outcome, we present three regression models: 1) unadjusted, 2) adjusted for demographics and baseline CVD risk (adjusted for age, sex, race/ethnicity, BMI, and self-reported hypertension, hyperlipidemia, or diabetes at Wave III), and 3) fully adjusted (adjusted for age, sex, race/ethnicity, household income, highest education, smoking, alcohol, AAS, weightlifting, exercise, strength training, individual sports, team sports, BMI, and self-reported hypertension, hyperlipidemia, or diabetes at Wave III). Wave III and Wave IV measures of smoking, alcohol, strength training, individual sports, and team sports were included in the fully adjusted model. Analyses were conducted using Stata 15.1 (StataCorp, College Station, TX).

#### Power calculations

Given a sample size of legal PES users (n = 963) and non-users (n = 11,033), and using an estimated mean for i) BMI change (Wave III to IV): 2.5±4.3 kg/m^2^, and ii) BMI (Wave IV): 29.0±7.5 kg/m^2^, our study had statistical power (alpha = .05, two-sided) of >80% to detect a i) 0.4 kg/m^2^ group difference in BMI change and ii) 0.7 kg/m^2^ group difference in BMI at Wave IV. Using an estimated mean for i) hemoglobin A1c 5.59±0.80%, ii) systolic blood pressure of 125.02±13.69 mmHg, iii) diastolic blood pressure of 79.37±10.21 mmHg, and iv) cholesterol deciles of 5.44±2.88, our study had statistical power (alpha = .05, two-sided) of >80% to detect a i) 0.08% group difference in hemoglobin A1c, ii) 1.28 mmHg group difference in systolic blood pressure, iii) 0.96 mmHg group difference in diastolic blood pressure, and iv) 0.27 group difference in cholesterol deciles. Given a sample size of legal PES users (n = 963) and non-users (n = 11,033), and using an estimated i) 6.5% with diabetes, ii) 51.9% with hypertension, and iii) 16.6% with hyperlipidemia, our study had statistical power (alpha = .05, two-sided) of >80% to detect odds ratios of i) 1.21 for hypertension, ii) 1.28 for hyperlipidemia, and iii) 1.41 for diabetes, respectively.

## Results

Overall, the 11,996 participants included in the analysis were on average 22 years of age at Wave III and were racially and ethnically diverse. Comparisons of descriptive and health characteristics of the sample are presented in Table 1, organized by legal PES use versus no use. In unadjusted comparisons, legal PES users were more likely to be White, male, college-educated, drink alcohol, and engage in weightlifting, exercise, individual sports, team sports, and other strength training than non-users. Legal PES users had higher parental household income than non-users. Frequency of health behaviors such as alcohol, smoking, and physical activity remained relatively stable over seven-year follow-up, except for the number of times participating in team sports, which decreased over follow-up. There were no significant differences in BMI or self-reported diabetes, hypertension, or hyperlipidemia diagnoses between PES users and non-users at Wave III. During the seven-year follow-up period, prevalence of diabetes, hypertension, and hyperlipidemia increased.

**Table 1:** Descriptive and health characteristics of 11,996 young adult participants in the National Longitudinal Study of adolescent health.

	Legal PES Use	No legal PES Use	
N	963	11,033	
**Demographic characteristics** (Wave III, 18–26 years)	Mean ± SE / %^a^	Mean ± SE / %^a^	p
Age, years	21.7 ± 0.1	21.8 ± 0.1	0.523
Sex			<0.001
Male	93.2%	46.8%	
Female	6.8%	53.3%	
Race/ethnicity			<0.001
White (non-Hispanic)	77.0%	67.6%	
Black/African American (non-Hispanic)	9.1%	16.0%	
Hispanic/Latino	9.9%	12.0%	
Asian/Pacific Islander (non-Hispanic)	2.5%	3.1%	
American Indian/Native American	0.6%	3.2%	
Other	0.9%	0.8%	
Household income, US dollars (Wave I, 11–18 years old)	52.7 ± 2.1	45.4 ± 1.4	<0.001
College education or more (Wave IV, 24–32 years old)	82.8%	73.6%	<0.001
**Health behaviors** (Wave III, 18–26 years old)			
Alcohol use, ≥2 days in the past month	69.2%	43.9%	<0.001
Smoking, past 30 days	38.7%	35.5%	0.181
Weight lifting to bulk up, past 7 days	33.6%	6.0%	<0.001
Exercise to bulk up, past 7 days	29.3%	6.4%	<0.001
Number of times participated in individual sports (running, wrestling, swimming, cross-country skiing, cycle racing, or martial arts), past 7 days	1.2 ± 0.1	0.6 ± 0.0	<0.001
Number of times participated in strenuous team sports (football, soccer, basketball, lacrosse, rugby, field hockey, ice hockey), past 7 days	0.9 ± 0.1	0.4 ± 0.0	<0.001
Number of times participated in gymnastics, weight lifting, or strength training, past 7 days	2.6 ± 0.1	0.8 ± 0.0	<0.001
Anabolic androgenic steroid use, between Wave I and III	13.7%	0.80%	<0.001
**Health behaviors** (Wave IV, 24–32 years old)			
Alcohol use, ≥2 days in the past month	68.8%	46.1%	<0.001
Smoking, past 30 days	38.3%	37.6%	0.789
Number of times participated in individual sports (running, wrestling, swimming, cross-country skiing, cycle racing, or martial arts), past 7 days	1.2 ± 0.1	0.7 ± 0.0	<0.001
Number of times participated in strenuous team sports (football, soccer, basketball, lacrosse, rugby, field hockey, ice hockey), past 7 days	0.5 ± 0.0	0.3 ± 0.0	<0.001
Number of times participated in gymnastics, weight lifting, or strength training, past 7 days	2.0 ± 0.1	0.8 ± 0.0	<0.001
**Physical health factors** (Wave III, 18–26 years)			
Body mass index	26.2 ± 0.2	26.5 ± 0.1	0.176
Diabetes, self-report	1.1%	0.9%	0.774
Hypertension, self-report	6.1%	5.5%	0.626
Hyperlipidemia, self-report	4.0%	4.3%	0.746
**Physical health factors** (Wave IV, 24–32 years old)			
Body mass index	28.7 ± 0.2	29.1 ± 0.2	0.230
Diabetes, self-report	2.1%	2.7%	0.323
Diabetes, self-report, hemoglobin A1c, medications	5.9%	6.6%	0.474
Hemoglobin A1c	5.6 ± 0.0	5.6 ± 0.0	0.802
Hypertension, self-report	14.9%	10.5%	0.007
Hypertension, self-report, blood pressure, medications	58.0%	51.3%	0.012
Systolic blood pressure	128.8 ± 0.6	124.7 ± 0.2	<0.001
Diastolic blood pressure	80.2 ± 0.5	79.3 ± 0.2	0.058
Hyperlipidemia, self-report	8.6%	8.2%	0.778
Hyperlipidemia, self-report, labs, medications	14.8%	16.7%	0.256
Total cholesterol decile	5.5 ± 0.1	5.6 ± 0.1	0.836
LDL cholesterol decile	5.6 ± 0.2	5.6 ± 0.1	0.986
HDL cholesterol decile	4.9 ± 0.1	5.5 ± 0.1	<0.001
Triglyceride decile	5.9 ± 0.1	5.6 ± 0.1	0.015

PES = performance-enhancing substance (such as creatine monohydrate or andro)

^a^All means and percentages are calculated with weighted data to reflect the representative proportion in the target US population.

Table 2 presents linear regression models with legal PES use as the independent variable and continuous CVD risk factors at seven-year follow-up as the dependent variable. In unadjusted linear regression models, legal PES use was associated with higher systolic blood pressure, a lower HDL cholesterol decile, and a higher triglyceride decile. In models adjusting for demographics, health behaviors, and Wave III CVD risk, there were no significant prospective associations between legal PES use and BMI, hemoglobin A1c, systolic blood pressure, and cholesterol (total, HDL, LDL, triglycerides) deciles at seven-year follow-up except for diastolic blood pressure. Legal PES use was associated with lower diastolic blood pressure in partially adjusted models, but the association was attenuated and was no longer statistically significant after adjusting for behavioral covariates. Table 3 presents logistic regression models with legal PES use as the independent variable and dichotomous CVD risk factors at seven-year follow up as the dependent variable. There were no significant prospective associations between legal PES use and diabetes, hyperlipidemia, or obesity based on objective measures or self-reported medications and diagnoses, adjusting for demographics, health behaviors, and Wave III CVD risk at seven-year follow-up. Legal PES use was associated with lower odds of hypertension in partially adjusted models, but the association was attenuated and was no longer statistically significant after adjusting for behavioral covariates.

**Table 2:** Associations between legal performance-enhancing substance use and continuous cardiovascular disease risk factors among young adults at seven-year follow-up.

Seven-year follow-up outcomes (ages 24–32 years)	Unadjusted		Adjusted, demographics^a^		Fully adjusted^b^	
Cardiovascular disease risk factors	B (95% CI)	p	B (95% CI)^a^	p	B (95% CI)^b^	p
Hemoglobin A1c	-0.01 (-0.08–0.06)	0.802	-0.04 (-0.11–0.02)	0.194	0.02 (-0.07–0.10)	0.669
Systolic blood pressure	4.15 (2.96–5.34)	<0.001	-0.49 (-1.69–0.72)	0.427	0.13 (-1.15–1.41)	0.838
Diastolic blood pressure	0.90 (-0.03–1.84)	0.058	-1.38 (-2.36 - -0.41)	0.006	-0.69 (-1.73–0.36)	0.197
Total cholesterol decile	0.03 (-0.32–0.26)	0.836	-0.15 (-0.45–0.14)	0.306	-0.15 (-0.46–0.17)	0.358
LDL cholesterol decile	0.00 (-0.32–0.32)	0.986	-0.11 (-0.43–0.21)	0.503	-0.14 (-0.49–0.21)	0.424
HDL cholesterol decile	-0.54 (-0.81 - -0.28)	<0.001	-0.02 (-0.29–0.25)	0.885	-0.15 (-0.43–0.14)	0.312
Triglyceride decile	0.32 (0.06–0.58)	0.015	-0.26 (-0.53–0.01)	0.059	-0.07 (-0.37–0.24)	0.665
Body mass index	-0.33 (-0.88–0.21)	0.230	-0.01 (-0.36–0.34)	0.958	0.18 (-0.19–0.55)	0.329
Body mass index change	0.00 (-0.36–0.35)	0.990	0.01 (-0.37–0.38)	0.971	0.15 (-0.26–0.56)	0.468

^a^Adjusted for age, sex, race/ethnicity, and BMI, hypertension, hyperlipidemia, or diabetes at Wave III.

^b^Adjusted for age, sex, race/ethnicity, household income, highest education, smoking,* alcohol,* anabolic-androgenic steroid use, weightlifting, exercise, strength training,* individual sports*, team sports*, BMI, and hypertension, hyperlipidemia, or diabetes at Wave III. * indicates adjusted for measures at both Wave III and Wave IV.

**Table 3:** Associations between legal performance-enhancing substance use and binary clinical cardiovascular disease risk factor outcomes among young adults at seven-year follow-up.

Seven-year follow-up outcomes (ages 24–32 years)	Unadjusted		Adjusted, demographics^a^		Fully adjusted^b^	
Cardiovascular disease risk factors	Odds ratio (95% CI)	p	Odds ratio (95% CI)^a^	p	Odds ratio (95% CI)^b^	p
Diabetes	0.88 (0.62–1.26)	0.496	0.94 (0.66–1.34)	0.713	1.37 (0.93–2.02)	0.107
Hypertension	1.31 (1.06–1.62)	0.012	0.78 (0.62–0.97)	0.027	0.86 (0.68–1.10)	0.232
Hyperlipidemia	0.86 (0.67–1.12)	0.276	0.83 (0.62–1.10)	0.198	0.94 (0.70–1.26)	0.676
Obesity	0.93 (0.78–1.11)	0.414	1.08 (0.87–1.34)	0.505	1.25 (0.98–1.59)	0.069

^a^Adjusted for age, sex, race/ethnicity, and BMI, hypertension, hyperlipidemia, or diabetes at Wave III.

^b^Adjusted for age, sex, race/ethnicity, household income, highest education, smoking,* alcohol,* anabolic-androgenic steroid use, weightlifting, exercise, strength training,* individual sports*, team sports*, BMI, and hypertension, hyperlipidemia, or diabetes at Wave III. * indicates adjusted for measures at both Wave III and Wave IV.

## Discussion

In this nationally representative cohort study of nearly 12,000 young adults in the US, we did not find significant prospective associations between legal PES use and CVD risk factors, including blood pressure, diabetes, lipids, and BMI, when adjusting for theoretically relevant covariates. Legal PES use was more common among males and participants with White race, higher education, higher income, alcohol use, and engagement in weightlifting, exercise, individual sports, team sports, and other strength training. Given the pervasive use of legal PES and current proposed legislation regarding the sale of legal PES to minors, research on long-term health effects of legal PES are warranted, especially given the paucity of studies and the lack of regulation by the Food and Drug Administration.

Our findings are consistent with earlier empirical research suggesting that legal PES use has few harmful effects on key risk factors for CVD. Previous research indicates that short- and long-term engagement of common PES, such as creatine and pre-workout supplements, do not pose a risk with respect to blood lipid profiles or hemodynamic properties [21, 51]. Our study extended these prior results, which were limited to a small sample of athletes, to investigate health outcomes of legal PES users over a longer time period in a much larger, general population sample without medical oversight. Additionally, although previous research has found an association between creatine use and weight gain [23], we did not find such a link, even in unadjusted analyses. This lack of association may provide a primary explanation for why we did not find an association between legal PES use and CVD risk, as weight and BMI are primary risk factors for CVD [24]. This may be in part due to our longer seven-year follow-up period, as well as the dichotomous operationalization of legal PES use and the overall broader conceptualization of legal PES that was not restricted to creatine only.

Additionally, our study adjusted for a large number of theoretically salient sociodemographic, behavioral, and health-related covariates, which were not accounted for in previous research. For example, Or and colleagues only controlled for age and gender [11], whereas in our study, the significant prospective associations between legal PES use and CVD risk that were found in unadjusted models were no longer significant when adjusting for Wave III CVD risk, broader sociodemographics, and other relevant behavioral variables. Thus, the association between legal PES and medical outcomes may be an indirect one, accounted for by the propensity of legal PES users to engage in other risky behaviors and to develop substance use and mental disorders. Further, Or and colleagues’ comparison group was composed of vitamin users, a subset of the population that have been shown to exhibit better health outcomes compared to the general population [52, 53]. Thus, the pro-health behaviors in that population may have contributed to the significant differences detected in that study moreso than the deleterious effects of PES. Finally, it is possible that the association between PES use and severe medical events may not be specifically related to CVD risk factors and may instead be the result of deleterious impacts on other organ systems (e.g., renal dysfunction [23]).

### Strengths and limitations

There are several limitations regarding the assessment of legal PES use. First, legal PES use was assessed as one broad category. While some examples were provided (such as creatine or andro), the Add Health survey did not list all examples of legal PES, which could also include β-alanine, L-carnitine, energy drinks, or others. Specific types of legal PES, such as those containing surreptitious AAS, selective androgen receptor modulators (SARMs), or other anabolic substances that are technically legal but sold primarily over the internet, could have associations with CVD risk; however, we were unable to separately analyze distinct types of legal PES. Second, the legal PES measure (yes/no) did not assess frequency, amount, or duration of use. It is possible that detecting significant associations with the health-related outcomes assessed here would require more precise dose- and duration-specific data. This is consistent with the notion that lasting physiological effects of substance use, including AAS [42], are exacerbated with longer duration and higher amounts of use. Third, the Add Health survey did not collect legal PES data at Wave IV so information on the rate maintaining legal PES use over a seven-year period is not available. Further, the concurrent use of multiple PES (e.g., creatine, pre-workout, prohormones as part of a PES regimen) could also be more likely to be significantly associated with greater CVD risk. In the current study, short-term, infrequent, and/or minimal use was indistinguishable from long-term, frequent, and high levels of use, and this heterogentity likely limited our ability to detect significant effects. Future research should assess the type, dose, and frequency of legal PES use in relation to future CVD risk.

In addition to the limitations regarding the assessment of PES use described above, another potential limitation of the study includes the use of self-reported measures, which could lead to reporting bias. We attempted to mitigate this by incorporating self-report of a physician diagnosis and objective measures into the Wave IV outcomes (the objective measures were not recorded at Wave III). Further, although the follow-up period in this study was seven years, it is possible that the medical and physiological effects of PES use, particularly if prolonged, emerge after a longer period of time or in age groups older than the young adult range reflected in the current sample. Although we adjusted for a large number of potential confounders, it is possible there were unmeasured confounders we were not able to adjust for. For instance, while we adjusted for frequency of sports participation, Add Health did not collect data on physical activity intensity or duration. Despite these limitations, however, there were numerous strengths to this study. First, we investigated a large, nationally representative sample and addressed an under-researched topic with important policy implications. Additionally, we extended the existing literature in this area by accounting for numerous theoretically-important covariates related to CVD risk which have not typically been included in previous studies on this topic. Further, the long-term, prospective nature of the study design contributes to the literature that has often focused on only short-term or immediate effects.

## Conclusions

While our study found no significant prospective associations between legal PES use and CVD risk factors, there remains a need for ongoing research into the long-term health outcomes of legal PES use. As noted by Pope et al. (2014), it is unlikely that randomized controlled trials will be conducted to test the outcomes of these substances [38]. This is in part due to the lack of funding, the unregulated nature of PES, and the potential risks associated with administering supraphysiologic doses of PES in clinical trials. Therefore, despite certain limitations inherent to their design, longitudinal cohort studies will represent invaluable sources of future data on the outcomes associated with legal PES use. Further, it will be important to account for the many confounding factors that may either exacerbate or protect against any potential adverse health outcomes. This may include exercise duration and intensity, dietary intake and eating patterns, health care utilization, income, stress, medical and mental health history, other substance use or misuse, and family medical and mental health history. Future research should investigate both the benefits and risks associated with legal PES use across age groups, as well as explore outcomes relevant to other organ systems of the body, such as the renal and liver systems.

In sum, there is growing evidence that legal PES use is common among adolescents and young adults, particularly among boys and men [1, 2], and these substances are unregulated and easily accessible in the US [3]. Despite the null findings in this study, prior research indicated that adverse health [11] and social [10, 47] outcomes are associated with legal PES use and our unadjusted models indicated that legal PES use was associated with certain CVD risk factors after seven years; however, it was apparent that other sociodemographic and behavioral variables better explained this association, as the associations were no longer significant in the fully adjusted models. Amid the lack of federal policy and oversight in the US, state policies have been introduced that aim to regulate the sale of these substances to minors, as well as provide detailed labeling that outlines the lack of oversight and research on outcomes associated with their use [54, 55]. Given these policies, it is imperative that research be funded and conducted to provide data on the safety and health effects, both risks and benefits, of legal PES use in order to inform public policy. Lastly, implementation and evaluation science will be needed to assess the effectiveness of any policies passed into law.
